# Use of Venovenous Extracorporeal Membrane Oxygenation in Critically-Ill Patients With COVID-19

**DOI:** 10.3389/fmed.2020.614569

**Published:** 2020-12-10

**Authors:** Mathieu Jozwiak, Jean-Daniel Chiche, Julien Charpentier, Zakaria Ait Hamou, Paul Jaubert, Sarah Benghanem, Pierre Dupland, Ariane Gavaud, Frédéric Péne, Alain Cariou, Jean-Paul Mira, Lee S. Nguyen

**Affiliations:** ^1^Assistance Publique – Hôpitaux de Paris, Hôpitaux universitaires Paris-Centre, Hôpital Cochin, Service de Médecine Intensive Réanimation, Paris, France; ^2^Université de Paris, Paris, France; ^3^CMC Ambroise Paré, Research and Innovation, Neuilly-sur-Seine, France

**Keywords:** acute respiratory distress syndrome—ARDS, COVID-19, extracorporeal membrane oxygenation (ECMO), intensive care unit (ICU), mechanical ventilation

## Abstract

Acute respiratory distress syndrome (ARDS) related to Coronavirus disease (COVID-19) is associated with high mortality. It has been suggested that venovenous extracorporeal membrane oxygenation (ECMO) was suitable in this indication, albeit the effects of ECMO on the mechanical respiratory parameters have been scarcely described. In this case-series, we prospectively described the use of venovenous ECMO and its effects on mechanical respiratory parameters in eleven COVID-19 patients with severe ARDS. Implantation of ECMO occurred 6 [3–11] days after the onset of mechanical ventilation. At the time of ECMO implantation, all patients received neuromuscular blocking agents, three (27%) received inhaled nitric oxide and prone positioning was performed in all patients with 4 [3−5] sessions of PP per patient. Under ECMO, the tidal volume was significantly decreased from 6.1 [4.0–6.3] to 3.4 [2.5–3.6] mL/kg of predicted body weight and the positive end-expiratory pressure level was increased by 25 ± 27% whereas the driving pressure and the mechanical power decreased by 33 ± 25% and 71 ± 27%, respectively. The PaO_2_/FiO_2_ ratio significantly increased from 68 [58–89] to 168 [137–218] and the oxygenation index significantly decreased from 28 [26–35] to 13 [10–15]. The duration of ECMO was 12 [8–25] days. Nine (82%) patients experienced ECMO-related complications and the main complication was major bleeding requiring blood transfusions. Intensive care unit mortality rate was 55% but no patient died from ECMO-related complications. In COVID-19 patients with severe ARDS, venovenous ECMO allowed ultra-protective ventilation, improved oxygenation and should be considered in highly selected patients with the most severe ARDS.

## Introduction

From December 2019 in China, a worldwide pandemic with an emergent coronavirus SARS-CoV-2 is responsible for Coronavirus disease (COVID-19) (1). While most COVID-19 patients are asymptomatic or mildly symptomatic, 15–70% of hospitalized patients develop an acute respiratory distress syndrome (ARDS) with a mortality rate of nearly 60% (2–5).

Most of these patients develop severe ARDS that require administration of neuromuscular blocking (NMB) agents and prone positioning (PP). In COVID-19 patients, venovenous extracorporeal membrane oxygenation (ECMO) has been described as suitable (6–11), such as previously employed during past respiratory virus outbreaks (i.e., Middle East respiratory syndrome and H1N1) (12).

To date, the effects of ECMO on mechanical respiratory parameters in COVID-19 patients have been scarcely described (9). In addition, a vast majority of COVID-19 patients develop a procoagulant state due to marked inflammatory response, responsible for venous thromboses and/or pulmonary embolism (13–16), which in turn could induce thrombotic complications of ECMO and complicate the anticoagulation management of these patients (9). Finally, in the context of pandemic with limited ECMO services, patients with ARDS eligible for ECMO should be carefully selected (17–20).

Thus, the aim of this study was to describe venovenous ECMO use and its effects on mechanical respiratory parameters in COVID-19 patients with severe ARDS hospitalized in the intensive care unit (ICU) of a University hospital.

## Patients and Methods

### Patients

This prospective and descriptive single-center study was conducted in the 24-bed ICU of Cochin University hospital. The study was approved by the Ethics Committee of the Société de Réanimation de Langue Française (CE SRLF 20–72). All patients or next of kin were informed about the study and consented to participate.

We included all consecutive patients under mechanical ventilation with the following criteria inclusion: (i) presence of ARDS according to the Berlin definition (21), (ii) venovenous ECMO implantation, and (iii) positive real-time reverse transcriptase-polymerase chain reaction assay in nasal swabs or pulmonary samples there was no exclusion criteria.

### Ventilatory Settings and Measurements

All patients were initially mechanically ventilated (CARESCAPE R860, GE Healthcare, Chicago, Il, United States of America) in the volume assist-controlled mode or in the pressure regulated volume control mode. Tidal volume was set at 6 mL/kg of predicted body weight. Respiratory rate and the inspiratory/expiratory time ratio were adjusted to prevent hypercapnia and avoid dynamic intrinsic PEEP. The PEEP level was titrated to reach a maximum plateau pressure of 30 cmH2O with a maximum driving pressure of 15 cmH2O. The inspired fraction of oxygen (FiO_2_) was titrated to obtain a peripheral oxygen saturation ≥90% (22). An airway humidification system was used in all patients.

The driving pressure was calculated as plateau pressure—total PEEP. The compliance of the respiratory system was calculated as tidal volume/(plateau pressure – total PEP). The mechanical power was calculated as 0.098 × tidal volume × respiratory rate × peak pressure – driving pressure/2 (23). Peak pressure was considered equal to plateau pressure in pressure regulated volume control mode. Oxygenation index was calculated as (mean airway pressure × FiO_2_)/arterial partial oxygen pressure (PaO_2_).

All mechanical respiratory parameters were continuously monitored with dedicated software, with a refreshing rate of two per minutes (GE Healthcare Centricity, Chicago, Il, United States of America). The total number of longitudinal endpoints was above two million time points.

### ECMO Settings

All patients were cannulated in femoro-jugular or in femoro-femoral with 19F jugular cannula and 23F femoral cannula. The ECMO flow was set to obtain a ECMO flow/cardiac output >60% (24) and the sweep gas flow was titrated to reach a capnia <45 mmHg. The fraction of inspired oxygen in circuit (FmO_2_) was titrated to obtain arterial oxygenation ≥90% (25). All patients received curative anticoagulation with unfractionated heparin (with a blood target anti-Xa between 0.5 and 0.7 UI/mL) (9). Patients were weaned off ECMO when clinical and radiological improvement after a successful weaning test, as described in EOLIA trial (25).

### Data Collection and Study Design

Demographic characteristics, comorbidities, clinical, ECMO, and biological data, therapeutics as well as ICU clinical outcomes were collected and analyzed. All clinical and biological data were recorded at the time of ECMO implantation, under ECMO, and after ECMO weaning.

### Statistical Analysis

Since it was a case series study, no sample size calculation was performed *a priori*. Variables were summarized as median [interquartile range] or counts and percentages. Continuous variables at the time of ECMO implantation, under ECMO and after ECMO weaning were compared with a Friedman test and *post-hoc* tests with correction for repeated measurements were performed when necessary. A *p* < 0.05 was considered statistically significant. Statistical analysis was performed with MedCalc 11.6.0 (MedCalc, Mariakerke, East Flanders, Belgium).

## Results

### Study Population

Between the 1st of March and the 31th of August, 96 COVID-19 patients were admitted in our ICU and 11 (12%) required ECMO: seven (64%) were men and the sole immunocompromised patient was a pregnant woman. Before ECMO implantation, all patients received intravenous antibiotics, two (18%) received corticosteroids and four (36%) received specific treatments for COVID-19: lopinavir/ritonavir in one patient, hydroxychloroquine in one patient, remdesivir in one patient and tocilizumab in one patient. The other baseline characteristics of patients are shown in Table 1. The duration of mechanical ventilation was 38 [32–53] days and ICU length of stay was 43 [36–53] days. The ICU mortality rate was 55%. Among the six deceased patients, four (67%) died in a context of withholding/withdrawal decisions, one died from multiple organ failure and one patient died from cardiac arrest due to pulmonary embolism.

**Table 1:** Baseline characteristics of patients.

Age (years)	50 [38–59]
Sex M/F (*n*)	7/4
Body mass index (kg/m^2^)	30 [26–32]
Simplified Acute Physiology Score	65 [57–77]
Sequential Organ Failure Assessment score	13 [9–14]
Hypertension (*n*, %)	2 (18)
Diabetes mellitus (*n*, %)	2 (18)
Smokers (*n*, %)	2 (18)
Obesity (*n*, %)	5 (4)
Immunodepression (*n*, %)	1 (9)

*n = 11. Data are expressed as median [interquartile range] or number (%)*.

### ECMO Management

Implantation of ECMO occurred 13 [10–22] days after the onset of symptoms, 6 [3–11] days after the onset of mechanical ventilation and 6 [4–11] days after ICU admission. Four (36%) patients were secondary transferred in our ICU after ECMO implantation by the ECMO mobile team and one (9%) patient was initially supported with veno-arterial femoro-femoral ECMO for cardiac arrest due to pulmonary embolism, and thereafter supported with venovenous ECMO. Ten (91%) patients were cannulated in femoro-jugular.

At the time of ECMO implantation, four (36%) patients received norepinephrine with a dosage of 0.49 [0.12–0.82] μg/kg/min, all patients received NMB agents, three (27%) received inhaled nitric oxide (iNO) and PP was performed in all patients with 4 [3–5] sessions of PP per patient. Patients were ventilated with a PEEP level of 14 [13–18] cmH2O and FiO_2_ of 100 [73–100]%. The driving pressure was 15 [10–19] cmH2O and the PaO_2_/FiO_2_ ratio was 68 [58–89] (Table 2).

**Table 2:** Ventilatory and biological parameters at the time of ECMO implantation, under ECMO and after ECMO weaning.

	**At the time of ECMO implantation**	**Under ECMO**	**After ECMO weaning**	***p*-value**
**Ventilatory parameters**				
Tidal volume (mL/kg of predicted body weight)	6.1 [4.0–6.3]	3.4 [2.5–3.6]^$^	5.8 [5.2–6.6]^£^	<0.0001
Respiratory rate (/min)	25 [22–32]	14 [12–16]^$^	33 [31–34]^£^	<0.0001
Total PEEP (cmH2O)	14 [13–18]	18 [17–20]^$^	10 [10–14]^*^^,^*^$^*	<0.0001
FiO_2_ (%)	100 [73–100]	60 [46–60]^$^	42 [35–50]^*^	<0.001
Plateau pressure (cmH2O)	29 [28–32]	26 [25–28]^$^	28 [27–30]	0.02
Driving pressure (cmH2O)	15 [10–19]	8 [7–11]^$^	16 [13–20]^£^	0.001
Compliance of respiratory system (mL/cmH2O)	20 [18–34]	27 [19–32]	23 [19–27]	0.92
Mechanical power (J/min)	25 [17–42]	6 [2–8]^$^	29 [27–34]^£^	<0.0001
Oxygenation index	28 [26–35]	13 [10–15]^$^	9 [7–11]^*^	<0.0001
**Biological parameters**				
PaO_2_/FiO_2_ ratio	68 [58–89]	168 [137–218]^$^	185 [165–257]^*^	<0.0001
PaCO_2_ (mmHg)	58 [48–70]	44 [41–48]^$^	54 [48–60]^£^	0.02
Lactate (mmol/L)	1.6 [1.4–2.1]	1.8 [1.1–2.3]	1.1 [0.9–1.4]^*^^,^*^$^*	0.03
pH	7.35 [7.29–7.37]	7.44 [7.33–7.47]^$^	7.38 [7.32–7.40]^£^	0.04

*n = 11. Data are expressed as median [interquartile range]*.

*P-value for Friedman test*.

* *p < 0.05 after ECMO weaning vs. at the time of ECMO implantation*.

£ *p < 0.05 after ECMO weaning vs. under ECMO*.

$ *p < 0.05 under ECMO vs. at the time of ECMO implantation*.

*ECMO, extracorporeal membrane oxygenation; PEEP, positive end-expiratory pressure; FiO_2_, inspired fraction of oxygen; PaO_2_, arterial partial oxygen pressure; PaCO_2_, arterial partial carbon dioxide pressure*.

The ECMO flow was 5.3 [5.0–6.0] L/min, the sweep gas flow was 7 [5–8] L/min and the FmO_2_ was 60 [52–80]%. Under ECMO, all patients received NMB agents, no patient received iNO and PP was performed in six (55%) patients with 4 [2–6] sessions of PP per patient.

The duration of ECMO was 12 [8–25] days and three (27%) patients died under ECMO in a context of care withdrawal. After ECMO weaning, four (50%) patients received NMB agents, no patient received iNO and PP was performed in three (38%) patients with 3 [2–5] sessions of PP per patient. The delay between ECMO weaning and extubation was 24 [11–41] days.

### Effects of ECMO on Mechanical Ventilation and Oxygenation

**Figure 1:**
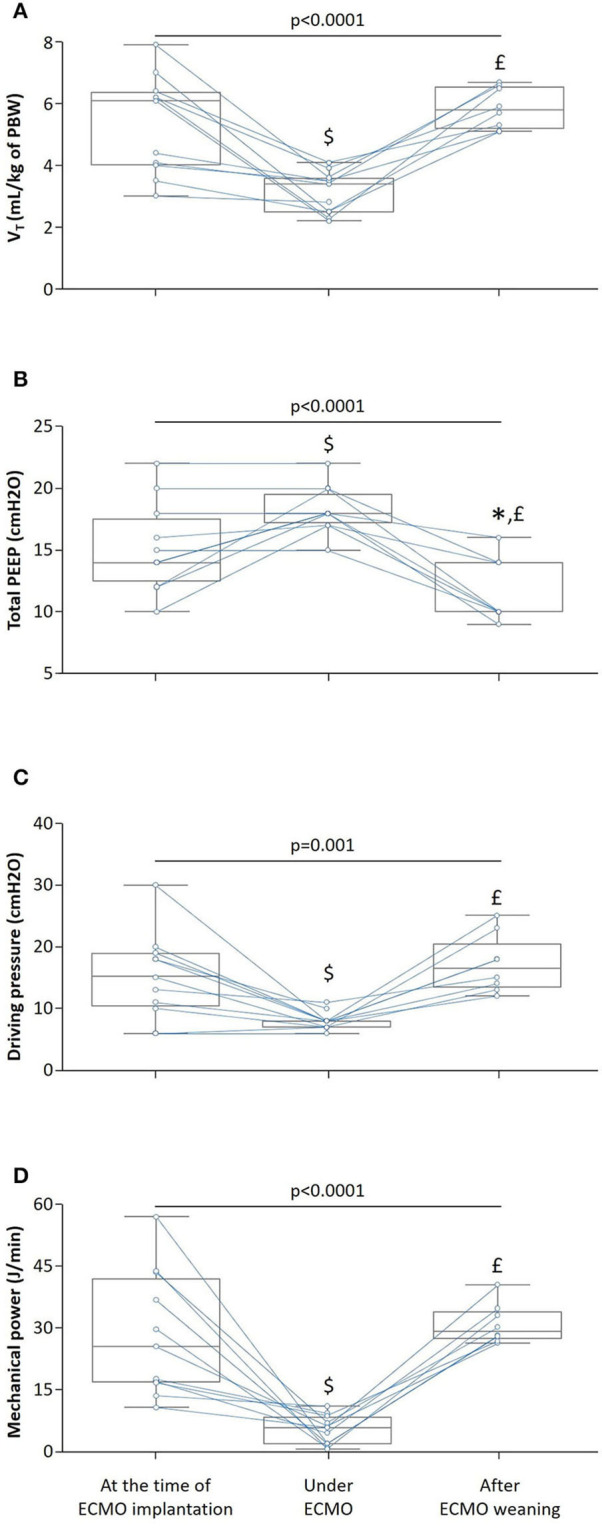
Effects of venovenous extracorporeal membrane oxygenation (ECMO) on mechanical ventilation. The box show the 25th and 75th percentiles, the line in the box the median and the whiskers the minimum and maximum values. Blue lines indicate individual changes. *n* = 11, *p*-value for Friedman test, *p < 0.05 after ECMO weaning vs. at the time of ECMO implantation, ^£^*p* < 0.05 after ECMO weaning vs. under ECMO and ^$^*p* < 0.05 under ECMO vs. at the time of ECMO implantation. **(A)** Tidal volume (V_T_). **(B)** Total positive end-expiratory pressure (PEEP). **(C)** Driving pressure. **(D)** Mechanical power. PBW, predicted body weight.

Under ECMO, the tidal volume was significantly decreased from 6.1 [4.0–6.3] to 3.4 [2.5–3.6] mL/kg of predicted body weight and the PEEP level was increased by 25 ± 27%. The driving pressure and the mechanical power decreased by 33 ± 25% and 71 ± 27%, respectively, whereas the compliance of the respiratory system remained unchanged (Table 2, Figure 1).

Under ECMO, the arterial partial carbon dioxide pressure decreased by 23 ± 19%, the PaO_2_/FiO_2_ ratio increased by 145 ± 77% and the oxygenation index significantly decreased from 28 [26–35] to 13 [10–15] (Table 2).

After ECMO weaning, oxygenation of patients was improved, as attested by a significant increase in PaO_2_/FiO_2_ ratio (185 [165–257] vs. 68 [58–89], *p* < 0.05) and a significant decrease in oxygenation index (9 [7–11] vs. 28 [26–35], *p* < 0.05), although the compliance of the respiratory system remained unchanged and the mechanical power increased (Table 2, Figure 1).

**Figure 2:**
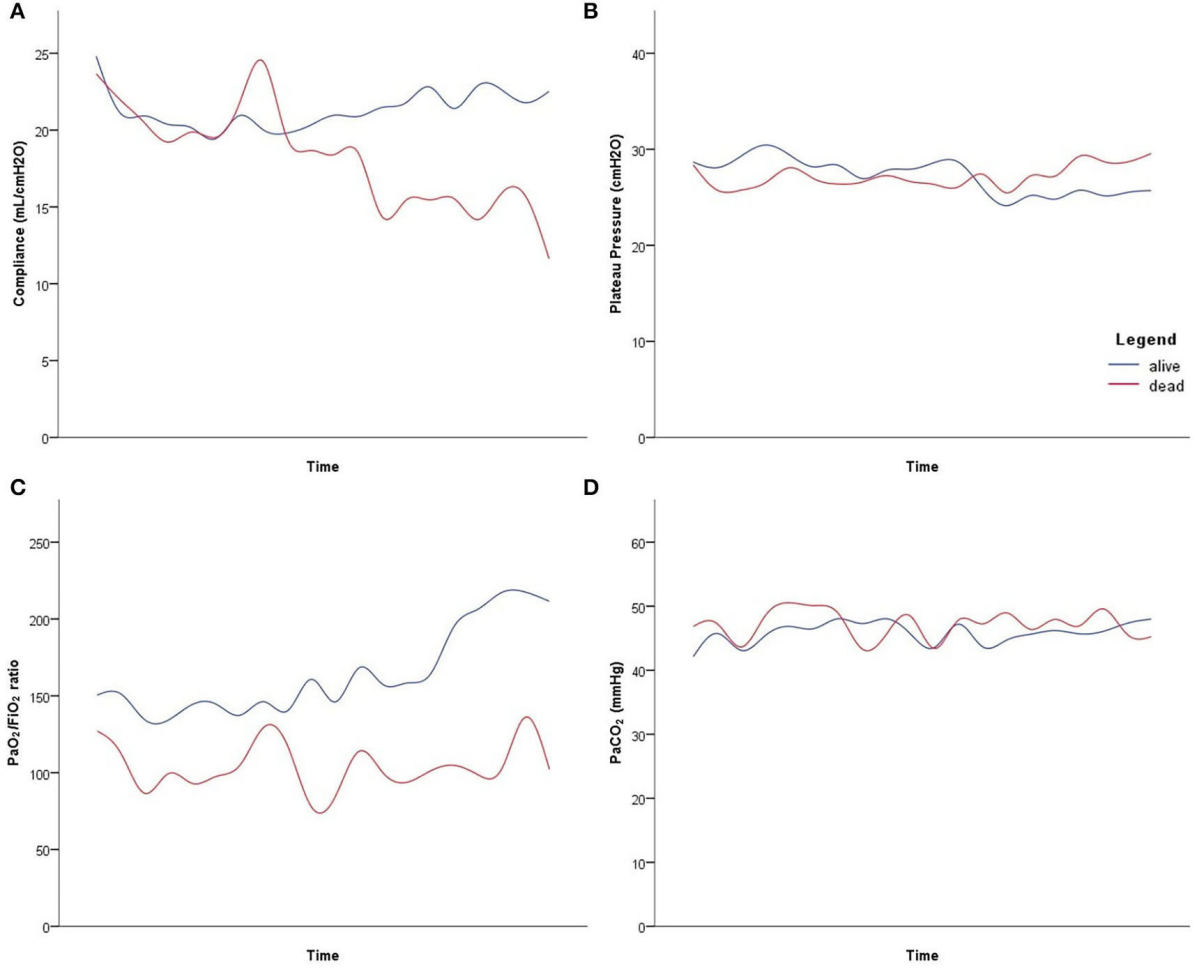
Evolution of the mechanical respiratory parameters under venovenous extracorporeal membrane oxygenation in deceased patients (red line) and in patients who were discharged alive from intensive care unit (blue line). Time was standardized for every patient, to have comparable x-axis graphical representation, with the first and last point representing the first and last value under extracorporeal membrane oxygenation, respectively. **(A)** Compliance of the respiratory system. **(B)** Plateau pressure. **(C)** Arterial partial pressure of oxygen/inspired fraction of oxygen (PaO_2_/FiO_2_) ratio. **(D)** arterial partial carbon dioxide pressure (PaCO_2_).

The evolution of the compliance of the respiratory system, plateau pressure, PaO_2_/FiO_2_ ratio and PaCO_2_ under ECMO in patients who died and those who survived the ICU time period is presented in Figure 2. Time was normalized and scaled for every patient to meet figure requirement (i.e., same x-axis) and to compare the evolution of these parameters between the two groups.

### ECMO-Related Complications

Nine (82%) patients experienced at least one ECMO-related complication: three had membrane or cannula thrombosis, three had major bleeding, three had pneumothorax, two had heparin-induced thrombocytopenia, two had intracranial bleeding (cerebellar hemorrhage or subarachnoid hemorrhage), two had cannula-associated deep vein thrombosis, two had major hemolysis and one patient had diffuse subcutaneous emphysema. No patient experienced pulmonary embolism due to ECMO and no patient died from ECMO-related complications.

Overall, eight (73%) patients required blood transfusions under ECMO: seven (64%) received red blood cell, six (55%) received platelet units and two (18%) patients received fresh-frozen plasma.

## Discussion

In this case series of COVID-19 patients with severe ARDS, our findings were as follows: (i) 12% of patients required venovenous ECMO with a high ICU mortality rate, (ii) ECMO allowed to decrease tidal volume and to increase PEEP level with a decrease in driving pressure and in mechanical power along with an oxygenation improvement, (iii) PP was necessary in more than half of the patients under ECMO and, (iv) the main ECMO-related complication was major bleeding requiring blood transfusions.

Venovenous ECMO might be useful in COVID-19 patients with severe ARDS (6–10), as previously described during past respiratory virus outbreaks (12). Here, we report the experience of our ICU of University hospital. In our cohort, 12% of patients required ECMO, a rate similar to that currently reported for COVID-19 patients (2, 26–28) and during the Middle East respiratory syndrome outbreak (29), but less than that reported for H1N1 pandemic (30–37). The duration of ECMO was 12 [8–25] days. Once again, or results confirmed the current literature in COVID-19 patients (9). In addition, the duration of ECMO we found was similar to that reported during H1N1 pandemic (32, 33, 35, 37) and the Middle East respiratory syndrome outbreak (29, 38).

The ICU mortality rate was high, confirming the poorer prognosis of patients requiring ECMO, both for COVID-19 patients (2, 7, 9, 39, 40) and during H1N1 (30–37) and the Middle East respiratory syndrome (41–44) pandemic, with mortality rate ranging from 40 to 100%. Interestingly, two recent retrospective and multicentric studies found that the estimated 60-days survival of ECMO was similar to that of non-COVID-19 patients (10) and that the estimated cumulative incidence of in-hospital mortality 90-days after the initiation of ECMO was 38% (11), highlighting the potential interest of ECMO in COVID-19 patients with severe ARDS. Nevertheless, it must be noted that the reported mortality rate in COVID-19 patients appeared to be higher than that reported during H1N1 pandemic (31, 32, 34–36). This could be in part explained by the fact that during H1N1 pandemic, ECMO was considered earlier than for COVID-19 pandemic, with shorter mechanical ventilation before ECMO implantation (32–34). Thus, because of the poor prognosis of these patients and because of limited ECMO services in this context of worldwide pandemic, COVID-19 patients with ARDS eligible for ECMO should be carefully selected (17–20). In this regard, some authors have proposed to take into account some additional criteria such as lymphocyte count and interleukin-6 blood level (45). Indeed, some studies have shown that both lymphopenia (2, 39) and increased interleukin-6 blood level (39) were prognosis factors in COVID-19 patients. However, ECMO may induce both a quantitative and qualitative decrease in lymphocyte count (46), but also an increase in interleukin-6 blood level (47). This latter was inversely correlated to mortality rate (47) and induced in animal models some lung parenchymal damage (48).

COVID-19 might lead to untypical ARDS with a discrepancy between marked hypoxemia but preserved lung compliance (49). Thus, despite diffuse alveolar damage in autopsy data, consistent with usual lung damage in patients with ARDS, some authors hypothesized that the physiopathology of ARDS related to COVID-19 might mainly resulted from the failure of the body's homeostatic O_2_-sensing system, and in particular, form an impairment of the hypoxic pulmonary vasoconstriction (50). In this regard, two opposite phenotypes of ARDS have been proposed: the “L-phenotype” for patients with nearly normal lung compliance and low lung recruitability in whom applying high PEEP level might be deleterious and the “H-phenotype” for patients with impaired lung compliance and high lung recruitability in whom applying high PEEP level could be beneficial (51). The variability of lung recruitability in COVID-19 patients with ARDS has been confirmed in small case series (49, 52–54). Interestingly, lung recruitability seemed difficult to predict and appeared to vary over time in these patients (52). Importantly, neither the compliance of the respiratory system nor the PaO_2_/FiO_2_ ratio could reliably predict lung recruitability and parameters such as the R/I ratio (i.e., the ratio between the compliance of the recruited lung and the compliance of the “baby lung”) might be used for this purpose (52).

So far, there is no data in the literature on the interest of ECMO according to the two types of phenotypes. In our cohort, ECMO allowed ultra-protective ventilation with a decreased tidal volume and respiratory rate, which in turn induced a decrease in driving pressure and mechanical power despite increased PEEP level. In addition, ECMO improved oxygenation, as attested by increased PaO_2_/FiO_2_ ratio and decreased oxygenation index. Our results are in agreement with those of Falcoz et al. (9). In addition, the effects of ECMO on mechanical ventilation are similar to those described in patients with ARDS non-related to COVID-19 (23). On an exploratory note, acknowledging the few patients, we observed in the longitudinal follow-up under ECMO, that patients who died seemed to benefit less from ECMO implantation than those who survived, particularly in regards to compliance of the respiratory system and oxygenation. Further analyses with more patients would be required with a similar follow-up including hundreds of thousands of time points, to comfort these observations. Finally, all patients under ECMO received NMB agents and PP was performed in 55% of patients. We confirmed that in COVID-19 patients, as in patients with ARDS non-related to COVID-19, NMB agents' administration and PP were the two most commonly used adjunctive therapies in patients under ECMO (23). It must be noted that in our cohort all patients received NMB agents at the time of ECMO implantation, compared to only 26% of patients with ARDS non-related to COVID-19 (23).

All but two patients (82% of patients) experienced ECMO-related complications, similar to ECMO-related complications in non-COVID-19 patients (23), but no patient died from ECMO-related complications. The main complication was major bleeding, including intracranial bleeding, requiring blood transfusions. Our results confirmed the first results in COVID-19 patients (9, 10), as well as results in patients with ARDS non-related to COVID-19, with 25% of them experiencing ECMO-related major bleeding requiring blood transfusions (23). Interestingly, the rate of thrombotic complications following ECMO in our cohort (18%) was lower than the rate of 71% recently reported by Parzy et al. (55). Several reasons may explain this discrepancy. Firstly, cannula-associated deep vein thrombosis appeared to be more frequent with femoral cannulation (55). However, all but one patient was cannulated in femoro-jugular. Secondly, cannula-associated deep vein thrombosis was investigated by CT-scan (55) and not by Doppler echography as in our cohort. Thirdly, due to a high incidence of venous thrombosis and pulmonary embolism in COVID-19 patients (13–16), levels of anticoagulation were likely higher than those in the study by Parzy et al. (55). Nevertheless, it must be noted the high variability of the rate of cannula-associated vein thrombosis in the literature, with a rate ranging from 18 to 85% (56–58).

We acknowledge some limitations to our study. First, this is a single-center study with small sample size, limiting statistical analysis. Indeed, because of the low number of patients, a global linear mixed model was not performed to compare deceased patients and patients who survived, hence a proper statistical comparison could not be performed between the two groups. Nevertheless, it should be noted that the figure represents the two groups well, with data aggregated from millions of measurements. Second, there was no available data regarding alveolar recruitment before ECMO implantation, in order to investigate the effects of ECMO according to the two different phenotypes of ARDS related to COVID-19. Third, the long-term respiratory function of these patients cannot be assessed at this time and should deserve further studies.

## Conclusion

In COVID-19 patients with severe ARDS, venovenous ECMO was a rescue therapy that allowed ultra-protective ventilation and improved patient oxygenation. However, because of the poor prognosis of these patients and because of limited ECMO services in this context of worldwide pandemic, COVID-19 patients with severe ARDS eligible for ECMO should be carefully selected in the light of benefit/risk balance in case of second pandemic wave.

## Data Availability Statement

The original contributions presented in the study are included in the article/supplementary materials, further inquiries can be directed to the corresponding author.

## Ethics Statement

The studies involving human participants were reviewed and approved by Ethics Committee of the Société de Réanimation de Langue Française. Written informed consent for participation was not required for this study in accordance with the national legislation and the institutional requirements.

## Author Contributions

MJ, J-DC, FP, AC, J-PM, and LSN conceived the study. MJ, JC, ZA, PJ, SB, PD, AG, and LSN recorded the data. MJ, J-DC, FP, AC, J-PM, and LSN analyzed data. All authors contributed to the interpretation of data, drafting the manuscript, and approved the final version of the manuscript. MJ and LSN drafted the first version of the manuscript.

## Conflict of Interest

The authors declare that the research was conducted in the absence of any commercial or financial relationships that could be construed as a potential conflict of interest.

## Abbreviations

ARDS: acute respiratory distress syndrome
ECMO: extracorporeal membrane oxygenation
FiO_2_: inspired fraction of oxygen
FmO_2_: fraction of inspired oxygen in circuit
ICU: intensive care unit
PP: prone positioning
PaO_2_: arterial partial pressure of oxygen
PEEP: positive end-expiratory pressure.
